# ACE-I Angioedema: Accurate Clinical Diagnosis May Prevent Epinephrine-Induced Harm

**DOI:** 10.5811/westjem.2016.2.29224

**Published:** 2016-04-26

**Authors:** R. Mason Curtis, Sarah Felder, Rozita Borici-Mazi, Ian Ball

**Affiliations:** * Western University, Division of Emergency Medicine, Department of Medicine, London, Ontario, Canada; † Queen’s University, Departments of Emergency Medicine and Biomedical and Molecular Sciences, Kingston, Ontario, Canada; ‡ Queen’s University, Division of Allergy and Immunology, Kingston, Ontario, Canada; § Queen’s University, Program in Critical Care Medicine, Kingston, Ontario, Canada; ¶ Western University, Division of Critical Care of Medicine, Department of Medicine, London, Ontario, Canada

## Abstract

**Introduction:**

Upper airway angioedema is a life-threatening emergency department (ED) presentation with increasing incidence. Angiotensin-converting enzyme inhibitor induced angioedema (AAE) is a non-mast cell mediated etiology of angioedema. Accurate diagnosis by clinical examination can optimize patient management and reduce morbidity from inappropriate treatment with epinephrine. The aim of this study is to describe the incidence of angioedema subtypes and the management of AAE. We evaluate the appropriateness of treatments and highlight preventable iatrogenic morbidity.

**Methods:**

We conducted a retrospective chart review of consecutive angioedema patients presenting to two tertiary care EDs between July 2007 and March 2012.

**Results:**

Of 1,702 medical records screened, 527 were included. The cause of angioedema was identified in 48.8% (n=257) of cases. The most common identifiable etiology was AAE (33.1%, n=85), with a 60.0% male predominance. The most common AAE management strategies included diphenhydramine (63.5%, n=54), corticosteroids (50.6%, n=43) and ranitidine (31.8%, n=27). Epinephrine was administered in 21.2% (n=18) of AAE patients, five of whom received repeated doses. Four AAE patients required admission (4.7%) and one required endotracheal intubation. Epinephrine induced morbidity in two patients, causing myocardial ischemia or dysrhythmia shortly after administration.

**Conclusion:**

AAE is the most common identifiable etiology of angioedema and can be accurately diagnosed by physical examination. It is easily confused with anaphylaxis and mismanaged with antihistamines, corticosteroids and epinephrine. There is little physiologic rationale for epinephrine use in AAE and much risk. Improved clinical differentiation of mast cell and non-mast cell mediated angioedema can optimize patient management.

## INTRODUCTION

### Background

Angioedema, defined as a transient, localized non-pitting edema of skin or mucous membranes is a potentially life-threatening emergency department (ED) presentation.^1^,^2^ Prompt identification of the cause of angioedema is essential, as it will guide management strategies and prevent the unnecessary and potentially harmful use of ineffective interventions. Etiologies may be broadly classified into mast cell mediated and non-mast cell mediated processes, the latter of which are thought to be mediated through accumulation of bradykinin.^3^ Bradykinin increases vascular permeability leading to plasma leakage into the dermis and resultant angioedema. This condition may be mediated by a hereditary or acquired etiology, the former of which is due to a deficiency in C-1 esterase inhibitor and presents with isolated angioedema without urticaria. Contrasting this, histamine release from mast cell mediated etiologies, such as an allergic response, leads to increased vascular permeability of more superficial layers of the dermis resulting in urticaria, and less commonly involves deeper dermal structures leading to angioedema.

Angiotensin-converting enzyme inhibitor (ACE-I) medications are reported to be responsible for approximately 30% of patients presenting to tertiary care and community EDs with angioedema.^4^,^5^ ACE-I induced angioedema (AAE), an example of an acquired form of non-mast cell mediated etiology of angioedema, is estimated to affect approximately 1 in 200 individuals taking an ACE-I and typically causes swelling of the lips, face and tongue, which has led to asphyxiation and death in severe cases.^6^,^7^ Although angioedema is considered a rare side effect of ACE-Is, the widespread use of this medication class, over 35–40 million individuals worldwide, means that a large number of individuals may be affected by this potentially life-threatening adverse effect.^8^

### Importance

ACE-Is are widely used for the management of hypertension, heart failure, remodeling after myocardial infarction, and for the prevention of diabetic nephropathy, cardiovascular events and secondary stroke. The incidence of AAE will continue to grow as the prevalence of cardiac disease and our average population age continue to rise.^9^

There are limited data on the optimal method for treating AAE, and the evidence and physiologic rationale supporting strategies such as epinephrine, corticosteroids and antihistamines is poor. Unnecessary exposure to these medications may be harmful, in particular epinephrine, as many of these patients are elderly and have ischemic heart disease. A greater awareness of appropriate therapeutic approaches to treat angioedema is required.^10^

### Goals of This Investigation

We sought to describe the presentation and management of angioedema in the ED, and highlight any epinephrine-induced harm. We highlight the need to differentiate mast cell and non-mast cell mediated etiologies of angioedema in order to optimize patient care and reduce iatrogenic morbidity.

## METHODS

### Study Population

We performed a retrospective chart review of all patients with angioedema presenting to two tertiary North American EDs between July 2007 and May 2012. Every patient visit with an International Classification of Disease, 10^th^ Revision discharge diagnosis of T782 (anaphylactic shock, unspecified), T783 (angioneurotic edema), T784 (allergy unspecified), D841 (defects in the complement system), T886 (anaphylactic shock due to adverse effect of correct drug or medicament) or T887 (unspecified adverse effect of drug or medicament) was eligible for review. Patient visits were excluded from the study if visible swelling was not documented on the medical record, if the medical evaluation was incomplete secondary to patient leaving against medical advice, or if the swelling was from a non-systemic reaction to an insect sting, trauma or irritant exposure.

### Methodology

Data abstraction was performed using a structured data extraction form. Training with the data extraction form was conducted on the first 20 medical records reviewed, which were then re-assessed during the formal review. Due to the binary nature of the abstracted data, such as the presence or absence of a documented clinical sign or symptom, or extraction of objective measures such as vitals, double data entry and inter-rater reliability were not assessed. This project was approved by the institutional review board at our academic center.

### Etiology

We assessed the most responsible etiology for angioedema based on the following a priori study definitions:

1. Food allergy: consumption of a known allergen in the two hours before presentation
2. Drug allergy: any change in drug regimen in the past 24 hours, except ACE-I or angiotensin receptor blocker (ARB)
3. Environmental allergy: exposure to a known or likely allergen (seasonal allergy, pets etc.)
4. Stinging insect: presentation of systemic response to known insect sting
5. ACE-I/ ARB induced: patients taking an ACE-I or ARB with no other known cause of angioedema.
6. Nonsteroidal anti-inflammatory drug (NSAID) or acetylsalicylic acid (ASA) induced: patients taking chronic NSAIDs or ASA with no other known cause of angioedema, who are not taking an ACE-I or ARB
7. C1-Esterase Inhibitor Deficiency: patients with known C1-esterase deficiency
8. Contact allergy: patients who present with a systemic reaction to contact with an allergen (i.e. cosmetic products, latex etc.)
9. Etiology unknown: patients whose presentation is not explained by any of the above definitions

These definitions have been previously used in similar studies.^4^,^11^ Patients using an ACE-I or an ARB were both defined as having AAE, as the mechanism behind ARB-induced angioedema has yet to be fully elucidated. This practice is consistent with previous studies.^12^,^13^

## RESULTS

### Study Population

**Figure 1:**
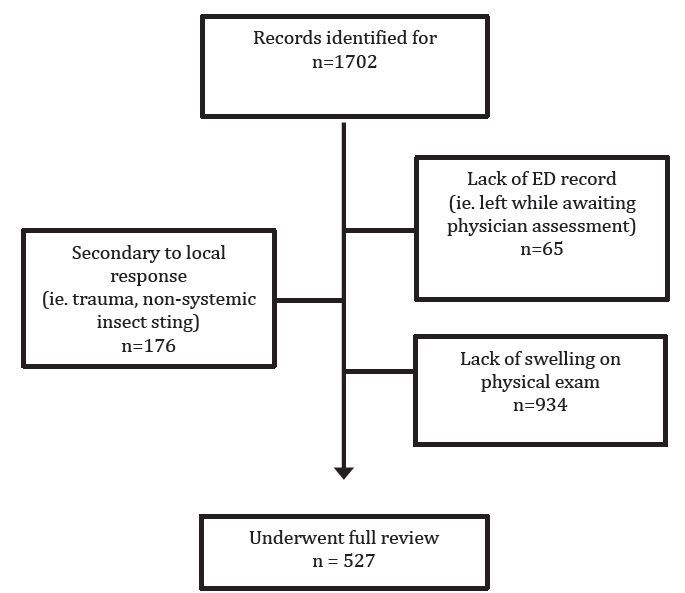
Flow chart of study population. *ED*, emergency department

**Figure 2:**
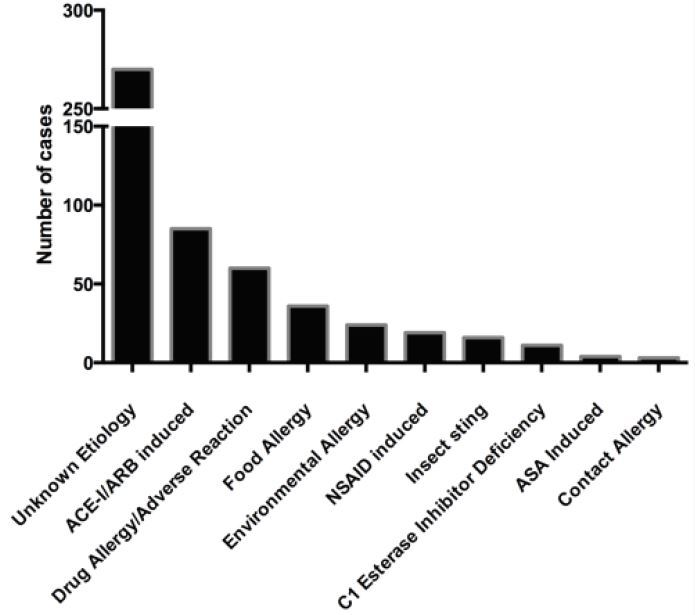
Graphical representation of angioedema etiologies in the emergency department. *ACE-I*, angiotensin-converting enzyme inhibitor; *ARB*, angiotensin II receptor blocker; *NSAID*, non-steroidal anti-inflammatory drug; *ASA,* acetylsalicylic acid

**Figure 3:**
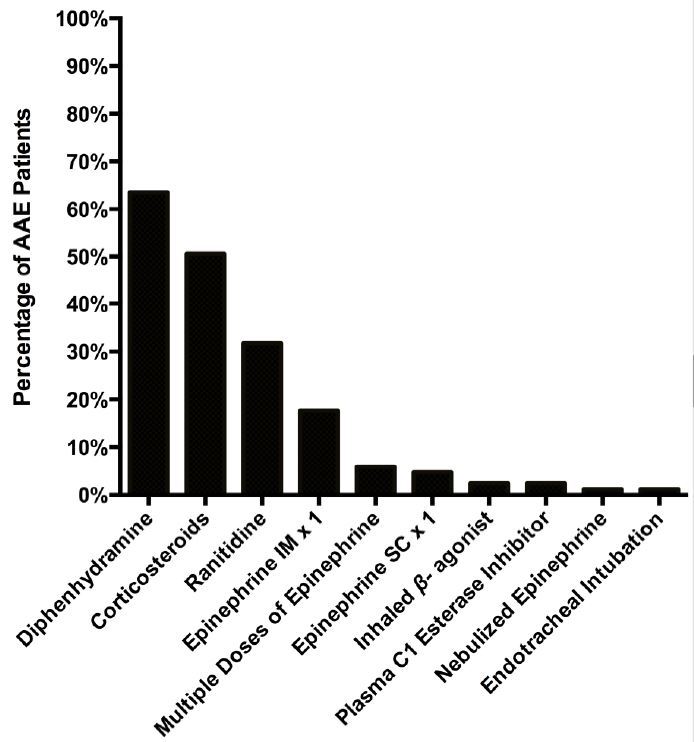
Management strategies employed in the emergency department for treatment of angiotensin-converting enzyme inhibitor induced angioedema (n=85). *IM,* intramuscular; *SC*, subcutaneous

Figure 1 describes the study population, representing a continuous sample of all patients presenting to the ED with angioedema during the five-year study period. Patient demographics and clinical variables are shown in Table. Figure 2 displays the likely presenting etiology, based on the *a priori* definitions. Notably, approximately 51% (n=270) had an unknown etiology, and the most common identifiable etiology was AAE (n=85, 33% of identifiable causes). Of the 85 AAE patients, ramipril was the most common ACE-I being used at the time of ED presentation (n=45, 53%). Seven of these patients were on an ARB at the time of ED presentation (8.2%). Figure 3 depicts the management strategies employed for patients presenting to the ED with AAE. (This figure does not include other causes of angioedema.) Antihistamines and corticosteroids were the most common strategies employed for AAE management. Of particular interest, epinephrine was administered through the subcutaneous or intramuscular route in 21% (n=18) of patients, five of whom received repeated doses.

### Epinephrine-Induced Morbidity

**Figure 4:**
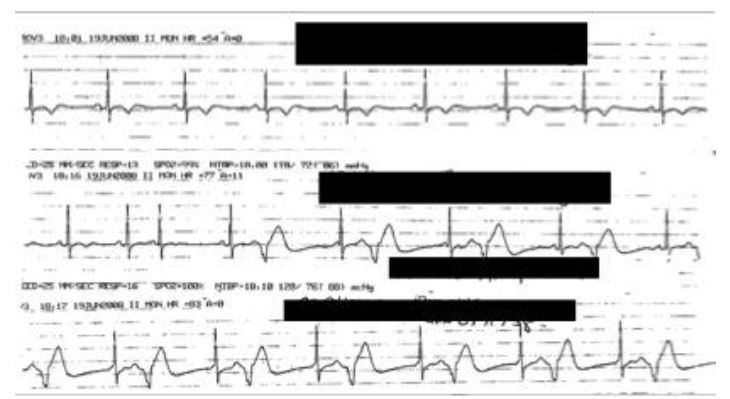
Rhythm strip of patient who developed ventricular bigeminy following epinephrine administration for treatment of angiotensin-converting enzyme inhibitor induced angioedema. Baseline rhythm shown in top line, post epinephrine dysrhythmia shown in middle and bottom line.

Epinephrine caused harm in two of 18 AAE patients who received it as management for AAE. In both cases, patients presented with isolated lingual edema with no signs of a mast cell mediated process (urticaria/pruritus, respiratory compromise, hypotension, nausea/vomiting). Both patients had been started on ramipril for cardioprotection following myocardial infarctions and were therefore particularly vulnerable to complications of epinephrine administration. One patient developed angina five minutes after epinephrine administration. The second patient developed runs of premature ventricular contractions after epinephrine administration, shown in Figure 4. There was no immediate improvement of the lingual edema after intramuscular epinephrine in either case. Angioedema symptoms slowly subsided over approximately five hours in both cases.

## DISCUSSION

We report that AAE is the most common identifiable cause of angioedema. Concerningly, our study found epinephrine, corticosteroids and diphenhydramine to be commonly used for the treatment of AAE. Of the 18 AAE patients who received epinephrine (21% of all AAE patients), five received multiple doses. This finding is not a local phenomenon, as other studies have identified that approximately 10–33% of AAE patients are being managed with epinephrine, some of whom receive repeated administrations.^4^,^11^,^12^ Cleary, epinephrine is still being used to manage AAE, despite its recognition as a non-mast cell mediated process.

In our study, two of the 18 AAE patients (11%) given epinephrine developed morbidity following intramuscular epinephrine administration. Given the high prevalence of epinephrine use in other studies for management of AAE, the two cases are unlikely to be unique.^4^,^11^,^12^ These patients may be some of the most vulnerable to complications from epinephrine administration, given their cardiac co-morbidities. As illustrated in the cases presented, the administration of epinephrine in patients with cardiac histories may cause ischemia, dysrhythmias, or complications thereof.

In the context of mast cell mediated angioedema, prompt administration of epinephrine is appropriate. It is well recognized that early administration of epinephrine for this form of angioedema, related to anaphylaxis, is the treatment of choice.^14^,^15^ The benefit is attributed to improved peripheral vascular tone, bronchodilation, reduction in mast cell mediated histamine release and positive inotropic and chronotropic effects on the myocardium.

In the acutely ill patient, when differentiation between a mast cell and non mast cell mediated etiology is difficult, we feel that clinicians should err on the side of giving epinephrine. In clear cases of AAE, however, clinicians should not administer epinephrine out of a need to do something. Removal of the offending medication, supportive care, and airway management are the appropriate approach in these scenarios.

To aid clinicians in the diagnosis of the systemic mast-cell mediated response that occurs in anaphylaxis, clinical guidelines have been established to guide physicians in its recognition and management.^16^ Sampson and colleagues have produced well known and widely accepted criteria for the diagnosis of anaphylaxis. These guidelines are based on clinical signs and stratified by potential exposures; if any of the following three criteria are met, anaphylaxis is likely and epinephrine should be administered promptly:

1. Acute onset of illness (minutes to hours) with dermal or mucosal involvement without known exposure, and either respiratory compromise, or reduced blood pressure
2. Exposure of a likely allergen with acute onset symptoms or signs in two organ systems (skin/mucosal tissue, respiratory compromise, reduced blood pressure, persistent gastrointestinal systems)
3. Exposure to a known allergen with reduced blood pressure (<90 mmHg systolic in adults, or >30% decrease in age specific systolic blood pressure).

When a patient’s presentation falls outside of these clinical criteria, it does not exclude anaphylaxis as a cause. However, in patients presenting with isolated lingual angioedema concurrently taking an ACE-I who do not meet these guidelines and have no urticaria, it is likely the etiology of their presentation is not driven by a mast-cell mediated mechanism.

Epinephrine administration for angioedema management outside the realm of anaphylaxis may cause significant morbidity and mortality. Previous case reports of the administration of epinephrine for non-anaphylaxis angioedema describe both cardiac dysrhythmias and ischemia, similar to what was observed in our study.^17^,^18^

Even when epinephrine is indicated in the setting of anaphylaxis, in a review of 166 admissions for management of anaphylaxis, Kanwar et al. found errors in dose and route of administration in 2.4% of patients, leading to coronary artery dissection, cardiogenic shock, coronary vasospasm and ventricular dysrhythmias requiring admission to critical care.^19^ This case series underscores the inherent risk of administering epinephrine and potentially lethal adverse outcomes that may arise. Given this risk of administration, combined with the unproven benefit of epinephrine in the setting of non-mast cell mediated angioedema, such as AAE, clinicians should not consider this an appropriate therapy for non-anaphylaxis angioedema. Avoiding the use of epinephrine and corticosteroids in the setting of hereditary and acquired angioedema is supported by consensus guidelines.^20^–^22^

### How Should We Manage AAE?

A recent review by Jaiganesh and colleagues highlights the need to differentiate mast cell and non-mast cell etiologies.^10^ They highlight that a focused assessment for the presence or absence of urticaria may aid clinicians in determining the etiology and appropriate therapeutic interventions. This distinction is key, as many mast cell mediated processes will present with urticaria secondary to histamine release. In contrast, the bradykinin mediated forms of angioedema, such as AAE and hereditary angioedema, are not associated with histamine release and urticaria; therefore, interventions such as antihistamines, corticosteroids and epinephrine offer limited therapeutic value based on underlying physiology.^6^

When a patient presents with angioedema, a prompt airway assessment is essential. Jaiganesh and colleagues describe a logical and effective step-wise approach to the patient with acute angioedema.^10^

Beyond airway management, treatment of AAE consists of supportive care (including potential endotracheal intubation) and discontinuation of the offending ACE-I. While seemingly obvious, discontinuation is necessary, for the recurrence of AAE is 10 times more likely when ACE-I are not discontinued.^23^ It is important to note that the duration of time between initiation of ACE-I therapy and onset of angioedema may be quite variable, reported to range from one day, up to eight years of therapy, with a median of six months.^24^ In the current study, no AAE patients received fresh frozen plasma or the bradykinin receptor antagonist icatibant, both of which have demonstrated potential benefit in small case series.^25^,^26^ In theory, the use of fresh frozen plasma would replenish angiotensin converting enzyme, and begin the breakdown of accumulated bradykinin; however, no prospective randomized trials have evaluated this and physicians must be prepared to deal with paradoxical exacerbation of symptoms.^21^ Contrasting this, icatibant is a bradykinin-receptor antagonist that has been used with good effect in the hereditary angioedema population, and recently been proposed for acquired cases of angioedema (such as in AAE). A recent randomized controlled trial of icatibant, for management of AAE demonstrated reduced time to symptom relief. 27 However, the small sample size, elevated cost of treatment and lack of impact on prognostic outcomes (need for intubation, need for surgical airway, prevention of admission), limit the generalizability of these results. A thorough review of these potential treatment modalities is nicely reviewed by Jaiganesh, and is found in many recent consensus guidelines and position statements, for the interested reader.^10^, ^20^–^22^

## LIMITATIONS

The retrospective nature of this investigation limits our data to the existing medical record. “Unknown Etiology” was grouped with all other causes of angioedema not due to an ACE-I. We did not perform double data entry and kappa analysis during data extraction. However, the binary nature (presence/absence) of extracted clinical variables limits the potential for bias.

## CONCLUSION

This descriptive analysis of angioedema in the ED identified AAE as the most common identifiable etiology of angioedema. Antihistamines and corticosteroids were the most frequently used ED therapies. Concerningly, epinephrine was often used despite a lack of evidence or physiologic rationale, and was responsible for documented morbidity in these high-risk patients. It is essential for physicians to distinguish between mast cell and bradykinin-mediated etiologies of angioedema, because their treatments differ. We do accept that epinephrine use is appropriate in undifferentiated cases. Unless new evidence of benefit from epinephrine use in AAE arises, epinephrine should be avoided for clear cases of non-mast cell mediated angioedema.

## Figures and Tables

**Table:** Demographics, clinical variables and pre-emergency department (ED) management of patients presenting to the ED with angioedema.

	AAE (n=85)	Non-AAE (n=442)
Mean age (SD)	65 (13)	33 (21)
Male sex, %	60	38
Swelling location, %		
Peri-orbital	2.4	38
Lips	40	42
Tongue	51	14
Cheek/face	15	21
Pharynx	17	11
Glottis	1.2	0
Extremities	3.5	11
Genitalia	1.2	1.1
Multiple sites	25	32
Time from onset (%)		
<1 hour	7.1	17
1–6 hours	59	38
6–24 hours	14	13
>24 hours	2.4	13
Pre-ED treatment (%)		
Antihistamine	24	34
Self Epi-Pen^®^	0	4.3
EMS epinephrine	3.5	3.4
EMS H_1_-blocker	5.9	2.5
Mean ED stay in minutes (SD)	238 (192)	198 (152)
Admission (%)	4.7	4.0
Intubation (%)	1.2	0.7

*AAE*, Angiotensin-converting enzyme inhibitor induced angioedema; *EMS,* emergency medical services
